# Evaluation of the Combined Effects of Stilbenoid from *Shorea gibbosa* and Vancomycin against Methicillin-Resistant *Staphylococcus aureus* (MRSA)

**DOI:** 10.3390/ph5091032

**Published:** 2012-09-20

**Authors:** Dayang Fredalina Basri, Chan Kin Luoi, Abdul Muin Azmi, Jalifah Latip

**Affiliations:** 1 School of Diagnostic and Applied Health Sciences, Faculty of Health Sciences, Universiti Kebangsaan Malaysia, Jalan Raja Muda Abdul Aziz, 50300 Kuala Lumpur, Malaysia; Email: chankinluoi@hotmail.com (C.K.L.); muinpmukm@gmail.com (A.M.A.); 2 Centre of Chemical Science and Food Technology, Faculty of Science and Technology, Universiti Kebangsaan Malaysia, Bangi, 43600 Bangi Selangor, Malaysia; Email: jali@ukm.my

**Keywords:** *Shorea gibbosa*, FIC, stilbenoid, bacteriostatic activity, additivity, MRSA

## Abstract

The aim of this study is to determine the combined effects of stilbenoids from *Shorea gibbosa* and vancomycin against methicillin-resistant *Staphylococcus aureus* (MRSA). A total of nine pure compounds, five stilbenoid dimers ε-viniferin, ampelopsin A, balanocarpol, laevifonol and diptoindonesin G and four stilbenoid trimers α-viniferin, johorenol A, ampelopsin E and vaticanol G were evaluated for their antibacterial activities against ATCC 33591 and a HUKM clinical isolate. Minimum inhibitory concentration (MIC) and minimum bactericidal concentration (MBC) for each active compound were determined using the serial microdilution and plate-streak techniques. The combined effect of stilbenoids with vancomycin against MRSA was evaluated using the checkerboard assay to determine their fractional inhibitory concentration (FIC) index values. The MIC value of α-viniferin on both MRSA strains was 100 μg/mL, whereas those of johorenol A on ATCC 33591 and HUKM strain were 100 μg/mL and 200 μg/mL, respectively. The MIC values of ampelopsin E and vaticanol G were higher than 400 μg/mL. Out of the five stilbenoid dimers, only ε-viniferin was capable of inhibiting the growth of both MRSA strains at MIC 400 μg/mL. The MBC value of ε-viniferin, α-viniferin and johorenol A showed bacteriostatic action against MRSA. The FIC index value of ε-viniferin and α-viniferin in combination with vancomycin showed an additive effect (0.5 < FIC ≤ 2.0) against both MRSA strains. Johorenol A-vancomycin combination was also additive against HUKM strain, but it showed synergistic interaction with vancomycin against ATCC 33591 (FIC < 0.5). Stilbenoid compounds from *Shorea gibbosa* have anti-MRSA activity and huge potential as an alternative phytotherapy in combating MRSA infections.

## 1. Introduction

*Staphylococcus aureus* is a type of normal microflora, which can be found on the skin and nose of a healthy person. However, this bacterium will become an opportunistic pathogen if it enters the body through a minor trauma or surgical wound in persons with compromised immune systems to cause skin and soft-tissue infections such as furunculosis and folliculitis [1]. *S. aureus* has become the major burn pathogen [2], as the primary isolate recovered in 75% of burn patients dying of septicaernia. Bowser-Wallace *et al.* [3] indicated that the incidence of invasive cultures also increased as burn size increased, with coagulase-positive *Staphylococcus* as the predominant invasive burn wound pathogenic isolate. In fact, S. *epidermidis* was the commonest organism within the first 24 post-operative hours, with *S.aureus* being the commonest isolate in the first week [4]. *S. aureus* is also the most important pathogen in diabetic foot infections, usually as a component of a mixed infection [5]. In patients with diabetes mellitus, *S. aureus* may be seen with increased frequency because of alterations in the mechanical barrier of the skin and vascular abnormalities common in people with diabetes [6].

Infections due to *S. aureus* have been treated with a range of antibiotics including penicillins, macrolides and aminoglycosides. However, this organism has successfully evolved numerous strategies for resisting the action of practically all antibiotics. The emergence of methicillin-resistant *Staphylococcus aureus* (MRSA) is causing major problems in the medical field as it has begun to resist other antibiotics [7]. In Malaysia, a 44.1% MRSA detection rate in both teaching and referral hospitals has been reported in Kuala Lumpur [8].

Vancomycin is now the last defense against MRSA infections, which are widely distributed in the community as well as in the hospital setting. In fact, intravenous vancomycin became the primary choice in treating MRSA infection. The most common side effect of vancomycin is Red Man syndrome, which can cause reddish skin and pruritus, a result of histamine reaction. Other related side effects are neutropenia, fever, phlebitis, nephrotoxicity, thrombocytopenia and Steven-Johnson syndrome [9]. Initially, MRSA isolates with reduced susceptibility to vancomycin or vancomycin-intermediate resistant *S. aureus* (VISA) were reported in Japan in 1996 [10]. However, the presence of vancomycin resistance in *S. aureus* isolates has threatened the efficacy of vancomycin in resisting MRSA [11], hence the need to develop alternative antibiotics for combating the problem of microorganism resistance.

**Figure 1:**
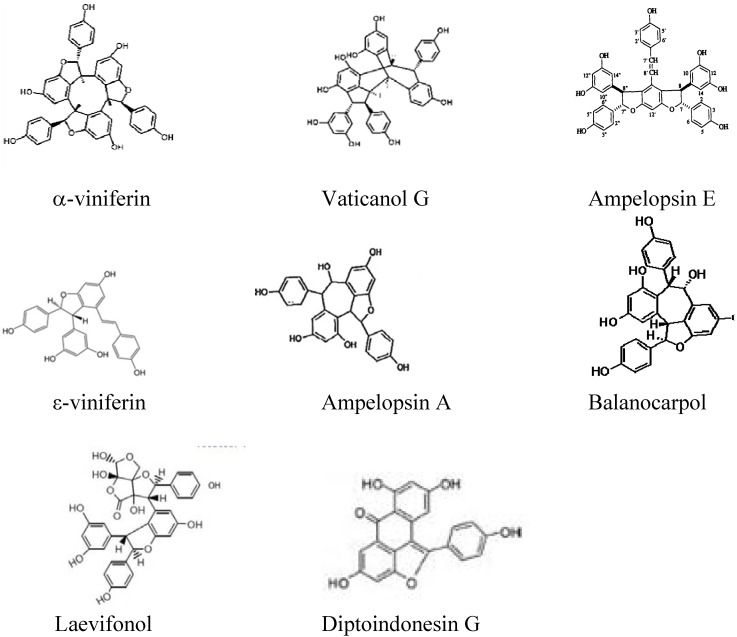
Chemical structures of stilbenoid trimers and dimers [21].

The drug-resistant bacteria are notably susceptible to plant-derived natural antimicrobials [12] and no doubt, antibacterial agents in plants have always been viewed as a source of novel therapies. *Shorea gibbosa* (also called Yellow Meranti) is a large emergent rainforest tree species of the Dipterocarpaceae family which is an excellent source of stilbenoid compounds belonging to the phenylpropanoid biochemical family. Phenylpropanoids are the largest group of secondary metabolites from plants. They can act as phytoalexins, antimicrobial agents in plants, by acting as a toxin to attack organism [13]. The main role of secondary metabolites is to protect the plants from being attacked by pathogens like virus, bacteria and fungi. They are important in the plant’s adaptation to the environment. Secondary metabolites from plants constitute a promising source of various types of bioactive chemicals having high capability to act as therapeutic agents [14]. Stilbenoids are formed in the flavonoid biosynthesis pathway. They are interesting natural products due to their role in resisting plant fungal pathogens [15]. Stilbenoid dimers and trimers in this study (Figure 1) belong to the group of stilbene oligomers. Stilbene oligomers have polyphenol functional groups and wide pharmacological applications [16] such as anti-inflammatory [17], antivirus [18], antibacterial [19] and antifungal [20] agents.

In the era of widespread of microbial resistance, multiple antibiotic combinations are needed to overcome infectious diseases [22]. Combining dual-function target sites of single antibiotics with antimicrobial agents will ensure the success of the treatment. To date, no prior literature has reported the effect of stilbenoids and their combinations with vancomycin on MRSA. In relation to this, a preliminary study was conducted to evaluate the potential of the interaction effect of stilbenoids and vancomycin on the growth inhibition of MRSA in the hope of obtaining synergistic interactions and hence, provide an option in the control of antibiotic resistance associated with nosocomial MRSA infections.

## 2. Results and Discussion

### 2.1. MIC of Stilbenoids and Vancomycin

The results of a total of nine stilbenoids which were evaluated for their anti-MRSA activity are shown in Table 1. The MIC values of ε-viniferin, α-viniferin and johorenol A ranged from 100 μg/mL–400 μg/mL. Out of the five dimer compounds studied, only ε-viniferin was capable of inhibiting both MRSA strains at MIC value 400 μg/mL whereas ampelopsin A, balanocarpol, laevifonol and diptoindonesin G were not capable of inhibiting the growth of MRSA at concentrations ranging from 400 μg/mL–0.78 μg/mL. As far as the stilbenoid trimers were concerned, the MIC values for α-viniferin against both MRSA strains were 100 μg/mL. The MIC value for johorenol A was also 100 μg/mL against MRSA ATCC 33591 however, HUKM strain demonstrated a lower susceptibility towards johorenol A with MIC value of 200 μg/mL. The MIC results of the other two stilbenoid trimers, ampelopsin E and vaticanol A, were higher than 400 μg/mL against both MRSA strains since they did not exhibit inhibitory effect against MRSA at concentrations ranging from 400 μg/mL–0.78 μg/mL. On the other hand, the MIC value of vancomycin against both MRSA strains was 2 μg/mL (Table 2) which is in agreement with the CLSI [23] susceptibility breakpoint for vancomycin against MRSA.

**Table 1:** Determination of MIC values of stilbenoid against ATCC 33591 and HUKM strain using the microbroth dilution technique.

Concentration (μg/mL)	ATCC 33591			HUKM strain		
	ε-Viniferin	α-Viniferin	Johorenol A	ε-viniferin	α-viniferin	Johorenol A
400	-	-	-	-	-	-
200	+	-	-	+	-	-
100	+	-	-	+	-	+
50	+	+	+	+	+	+
25	+	+	+	+	+	+
12.5	+	+	+	+	+	+
6.25	+	+	+	+	+	+
3.13	+	+	+	+	+	+
1.56	+	+	+	+	+	+
0.78	+	+	+	+	+	+

+: presence of red colour changes in the well (presence of growth); -: absence of red colour changes (absence of growth); positive control: bacterial suspensions and Mueller-Hinton broth; negative control: stilbenoids tested and Mueller-Hinton broth.

The only stilbenoid dimer which showed an inhibitory effect against MRSA was ε-viniferin. A previous study by Yim *et al.* [24] reported that *trans*-ε-viniferin from *Vitis amurensis* displayed the strongest activity against *S. mutans* and *S.sanguis* hence, this implies that this resveratrol dimer is active against mostly Gram-positive bacteria. Our finding showed that α-viniferin is a stronger anti-MRSA agent compared to ε-viniferin. This could be due to the trimeric structure of this resveratrol oligostilbene. This is in accordance with Zain *et al.*’s report [25] that *S. aureus* and *Escherichia coli* were susceptible to the effect of α-viniferin from *Dipterocarpus verrucosus*. In addition to Gram-positive bacteria, α-viniferin extracted from the brown-red wood of *Vitis vinefera* plays an important role in inhibiting fungal growth on grapevines [26]. It is believed that α-viniferin can inhibit modification of oxidative proteina, which was induced by copper ions, lipid peroxidation and superoxidative anion production [15].

**Table 2:** Determination of MIC values of vancomycin against ATCC 33591 and HUKM strain using microbroth dilution technique.

Concentration (μg/mL)	Vancomycin	
	ATCC 33591	HUKM strain
250	-	-
125	-	-
62.5	-	-
31.25	-	-
15.6	-	-
7.8	-	-
3.9	-	-
2	-	-
1	+	+
0.5	+	+

+: presence of red colour changes in the well (presence of growth); -: no colour changes in the well (absence of growth); positive control: bacterial suspensions and Mueller-Hinton broth; negative control: vancomycin and Mueller-Hinton broth.

It is interesting to report the activity of johorenol A against different strains of MRSA. Our results showed that MRSA ATCC strain appeared to be more susceptible than the clinical isolate towards the effect of johorenol A. This is a unique stilbenoid trimer because, until now, there is no literature on its biological activities. The fact that it’s anti-MRSA activity is comparable with α-viniferin against ATCC strain warrants a more thorough investigation of its chemical structure.

No antimicrobial activity was displayed by ampelopsin A and ampelopsin B, which is in line with Kundakovic *et al.* [27] who reported weak antimicrobial activity of extracts from *Ampelopsis brevipedunculata*. Laevifonol from the stem bark of *Vatica odorata* was also shown to possess weak antimicrobial activity against *S. aureus* compared to erythromycin [28]. On the other hand, diptoindonesin G from the stem bark of *Hopea chinensis* has been recommended as a starting molecule for the discovery of immunosuppressive agents [29], whereas balanocarpol and vaticanol G from the stem bark of *Hopea species* showed a more promising cytotoxic activity and antioxidant effect, respectively [30], rather than as antibacterial agents.

### 2.2. MBCs of Stilbenoids

As seen from Table 3, the MBC values of all the stilbenoids tested cannot be determined as all the samples from the clear wells from the microbroth dilution test showed bacterial growth after streaking on nutrient agar plates. This indicates that MBC values of ε-viniferin, α-viniferin and johorenol A could well be higher than their MIC values. It can therefore be interpreted that they act against the MRSA strains by a bacteriostatic action. The determination of such properties was based on the growth of small colonies of MRSA after the inoculum was subcultured on the antibiotic-free media. Other phenolic compounds such as tannin and curcumin were also considered as bacteriostatic [31,32]. This could imply that polyphenols exert their antimicrobial activity through their biostatic rather than their biocidal effects [33]. However, further investigation is warranted to evaluate the bacteriostatic mode of action of stilbenoids.

**Table 3:** Determination of MBC values of ε-vineferin, α-viniferin and johorenol A.

MIC (μg/mL)	ATCC 33591			MRSA HUKM		
	ε-Vineferin	α-Vineferin	Johorenol A	ε-Vineferin	α-Vineferin	Johorenol A
400	+	+	+	+	+	+
200	ND	+	+	ND	+	+
100	ND	+	+	ND	+	ND
50	ND	ND	ND	ND	ND	ND
25	ND	ND	ND	ND	ND	ND

+: growth of bacteria on NA plate; -: no growth of bacteria on NA plate; ND: not done because the microtiter well at the tested concentration showed the presence of bacterial growth as shown in Table 1.

### 2.3. FIC of Stilbenoids and Vancomycin

The results for the type of interaction effect between stilbenoid dimers and trimers with vancomycin are presented in Table 4, as determined by calculation of FIC index values. The FIC index of ε-viniferin in combination with vancomycin against both MRSA strains was 0.5625 *i.e*., greater than 0.5 but less than 2.0 (0.5 < x ≤ 2.0), indicating an additive interaction.

**Table 4:** Determination of FIC index values and outcome of interaction of stilbenoid/vancomycin combination against MRSA strains.

Strains	Agent	MIC (μg/mL)		FIC (μg/mL)		Outcome
		Alone	Combination	FIC	FICI^−^	
ATCC 33591	ε-viniferin	400	200	0.5	0.5625	Additive
	Vancomycin	1.5	0.0938	0.0625		
	α-viniferin	100	50	0.5	0.5625	Additive
	Vancomycin	2	0.125	0.0625		
	Johorenol A	100	6.25	0.0625	0.3125	Synergistic
	Vancomycin	2	0.5	0.25		
					
MRSA HUKM	ε-viniferin	400	200	0.5	0.5625	Additive
	Vancomycin	1.5	0.0938	0.0625		
	α-viniferin	100	100	1.0	1.0625	Additive
	Vancomycin	2	0.125	0.0625		
	Johorenol A	200	100	0.5	0.5625	Additive
	Vancomycin	2	0.125	0.0625		

As far as stilbenoid trimers are concerned, the combination of α-viniferin and johorenol A with vancomycin also displayed an additive effect (except for johorenol A against HUKM strain) against MRSA. Surprisingly, the FIC index of the latter was 0.3125, which was <0.5, indicating a synergistic interaction. Johorenol A reduced the MIC value of vancomycin by four-fold to produce the synergistic effect upon their combination on MRSA ATCC 33591. All the additive interactions showed a sixteen-fold decrease in the MIC of vancomycin against the MRSA strains studied.

An additive effect observed for the combinations in the microdilution checkerboard assay could possibly indicate interaction of the stilbenoids with common target residues similar to that of vancomycin, which lead to competitive inhibition and consequently, no inhibition of cell growth [34]. In another study involving epigallocatechin gallate (EGCg), cell wall damage was suggested as the main factor responsible for the additivity results in its combination with β-lactam antibiotics against MRSA [35]. The combination of *Quercus infectoria* extract with vancomycin, which resulted in additive mode of interaction against MRSA possibly suggest that these phytochemicals may act at the same target sites in the cytoplasmic membrane with that of vancomycin [36]. It has been reported that some plant-derived compounds can improve the *in vitro* activity of some cell-wall inhibiting antibiotics by directly attacking the same target site, *i.e*., peptidoglycans [37]. Vancomycin acts by inhibiting synthesis and assembly of growing bacterial cell walls, hence, changing bacterial cytoplasmic membrane permeability [38]. Polyphenolic compounds have also been shown to exert their antibacterial action through membrane perturbations [39].

Combination antimicrobial therapy may produce synergistic effects in the treatment of bacterial infections and has been shown to delay the emergence of antimicrobial resistance [40]. In this study, johorenol A is the only stilbenoid that exhibited a synergistic interaction with vancomycin against the ATCC strain. A possible suggestion could be that johorenol A potentiated the activity of vancomycin, giving rise to synergism. However, only the checkerboard technique was used to evaluate the combination effect in this study whereby, time-kill assay is also one of the common methods used in synergy testing. Although the latter is more labor-intensive and time-consuming, the checkerboard method merely reflected the inhibition of bacterial growth whereas the time-kill methodology measures the extent of killing [41]. Generally, synergistic effects are more often detected by using time-kill methods rather than the checkerboard method. If the combination effect proved to be synergistic in the checkerboard assay, they would also be expected to affect the time-kill assay synergistically. On the other hand, an additive outcome from checkerboard test does not necessarily correlate with additivity results in the time-kill test [42]. Since this is the first study on the effect of stilbenoids and its combination with vancomycin against MRSA, a time-kill study to confirm their interaction in exerting the bacteriostatic effect on this pathogen is recommended.

## 3. Experimental

### 3.1. Bacterial Strains

The bacterial strains used in the study were MRSA ATCC 33591 and HUKM strain. MRSA strain ATCC 33591 was obtained from the American Type Culture Collection (Manassas, VA, USA) whereas the HUKM strain was obtained and characterized from clinical samples of infected patients in Universiti Kebangsaan Malaysia Hospital, Kuala Lumpur. They were cultured on nutrient agar and incubated at 37 °C for 24 h. The colony formed was then inoculated onto the slanting agar in a bijou bottle and incubated at 37 °C for 24 h. The colony stock was kept at 4 °C to be used for three months from the date of preparation.

### 3.2. Antimicrobial Agents

Vancomycin in powder form was obtained from Sigma Aldrich (St. Louis, MO, USA). Stilbenoid dimers used in this study were ε-viniferin, ampelopsin A, balanocarpol, laevifonol and diptoindonesin G while α-viniferin, ampelopsin E, vaticanol G and johorenol A were the stilbenoid trimers, all of which were in the form of pure compounds extracted as active constituents from *Shorea gibbosa.* The initial concentration of vancomycin used in the study was 2,000 μg/mL whereas the concentration of all the stilbenoids tested was 800 μg/mL.

### 3.3. Bacterial Suspensions

Bacterial suspensions used in the determination of MIC and FIC were prepared by pre-culture dilution, where they were cultured on nutrient agar at 37 °C for 24 h before dilution. Two to five colonial isolates were inoculated into 5 mL Mueller-Hinton broth in a bijou bottle using a sterile wire loop. The broth culture was then incubated at 35 °C for 24 h. Turbidity of the bacterial suspension was adjusted to 0.5 McFarland or 1.5 × 10^8^ CFU/mL using sterile Mueller-Hinton (MH) broth, which corresponded to optical density (OD) value of 0.08 at the wavelength of 625 nm. The suspension with 0.5 McFarland was diluted again by MH broth with the ratio of 1:100 in order to get an inoculum size equivalent to 1–5 × 10^6^ CFU/mL.

### 3.4. MIC Determination

The MIC of stilbenoids and vancomycin on MRSA was determined by the standard microtitre broth dilution method [23]. The wells of 96-microtitre plate were filled with 50 µL Mueller-Hinton broth. About 50 µL of the tested stilbenoids or vancomycin at final concentration of 800 μg/mL or 2,000 μg/mL respectively, was then pipetted into the first well. Serial microdilution was done by drawing 50 µL mixture from the first well into the next one. This step was repeated until 12th well and the last 50 µL mixture was discarded. The tested solutions were sterilized using a 0.20 µm syringe filter. A volume of 50 µL bacterial suspension was pipetted into all the wells. Thus, there was a total of 100 µL mixture comprised of MH broth, antimicrobial agent and bacterial culture in each well. A mixture of MH broth and the tested compounds made up the negative control whereas the positive control contained mixture of MH broth and the inoculum. The 96-well plate was then covered by a sterile cover lid, sealed with parafilm before it was incubated at 37 °C for 24 h. The test was done in triplicate for each antimicrobial agent. After incubation, turbidity of each well was examined by adding 20 µL of 2,000 μg/mL 2,3,5-triphenyltetrazolium chloride (TTC) into each well and incubated at 37 °C for 30 min. Presence of red colour in the well showed bacterial growth while no colour changes in the well indicated no growth. The MIC value was defined as the lowest antimicrobial concentration which prevented visible bacterial growth [43].

### 3.5. MBC Determination

From each of the wells which is clear, about 10 µL aliquot was drawn and subcultured on nutrient agar. The plate plates were then incubated at 37 °C for 24 h. Bacterial growth on agar was observed and the concentration which has a colony count of less than 10 was considered as the MBC value. The MBC value was defined as the lowest concentration of antimicrobial agent that can kill >99% of the microorganism population where there is no visible growth on nutrient agar.

### 3.6. FIC Determination

The effects of interactions between stilbenoids and vancomycin against MRSA ATCC 33591 and HUKM strain were evaluated using the checkerboard technique. Determination of FIC index value is commonly used as a measurement of interactive inhibition using 96-well plates [44]. The concentration of stilbenoids and vancomycin were prepared in five concentrations, namely 1/16 × MIC, 1/8 × MIC, 1/4 × MIC, 1/2 × MIC and 1 × MIC. Along the x-axis across the checkerboard plate, 50 µL of each stilbenoid compound was added into each well in the following sequence: 1/16 × MIC, 1/8 × MIC, 1/4 × MIC, 1/2 × MIC and 1 × MIC. As for the y-axis, 50 µL vancomycin was added into each well in the same sequence as the tested stilbenoids. Inoculum size of approximately 5 × 10^5^ CFU/mL was then added into all the wells. The well containing MH broth and bacterial suspension served as positive control. On the other hand, negative control comprised only MH broth and the tested antimicrobial agents. The 96-well plate was then sealed and incubated at 37 °C for 30 min. The presence of red colour in the well indicated positive growth while no colour changes indicated negative results. The FIC index values was then calculated using the following formula:


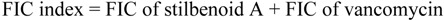


where:


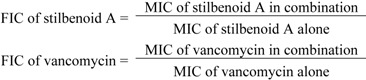


Synergistic effect was defined as an FIC index of 0.5 or less, additive effect as an FIC index of more than 0.5 and less than 2 and an antagonistic effect as an FIC index exceeding 4.0 [45].

## 4. Conclusions

This study revealed that the stilbenoind ε-viniferin, α-viniferin and johorenol A are bacteriostatic towards the growth of MRSA. All the combinations of these stilbenoids with vancomycin displayed additive effects except for johorenol A, which is found to act in synergism with vancomycin against MRSA ATCC 33591. Thus, active stilbenoid compounds offer huge potential as alternative phytotherapy against MRSA. However, the MIC, MBC and FIC evaluations are just an *in vitro* preliminary study to test the effectiveness of these compounds as antibacterial agents. The next focus should be on morphological and ultrastructural analysis of MRSA strains using scanning electron microscope (SEM) or transmission electron microscope (TEM) to observe the effects of these natural compounds with reference to vancomycin and when used in combination. Time-kill kinetic studies to evaluate their synergistic activity of stilbenoids from *Shorea gibbosa* in combination with vancomycinare under current progress in our laboratory.
